# New Method of Operating for Radical Cure of Inguinal Hernia

**Published:** 1851-03

**Authors:** M. Varette


### ARTICLE X.

### *New Method of Operating for the Radical Cure of Inguinal Hernia.* By M. VARETTE.

In a note to the French Academy of Sciences, by M. Varette, which was referred to a select commission, composed of Messrs. Velpeau and Lallemand, we find the following concerning the radical cure of inguinal hernia :

"I had the honor," said M. Varette, "to address to the Academy, in May and November of 1849, some observations on the radical cure of inguinal hernia, obtained by a method which appeared to me to be novel. I have not yet sufficient facts to treat of the matter in all its details; nevertheless, I hope the Academy will receive the announcement of an operation which I have conceived for the cure of an affection, which has as yet defied all the resources of art. This operation consists—firstly, in folding about the whole extent of the inguinal canal, and even beyond it, a tegumentary plug; secondly, in retaining it *in situ*, for a sufficient length of time; and, thirdly, in obtaining by means of cauterization, solid adhesions over a large surface."

M. Varette then gives a description of an apparatus for operation, the engraving representing which is not given by the Academy in its transactions, and consequently we should be unable to convey its merits fully by description. It is enough to say, however, that, the hernia being reduced, and the surgeon with his index finger, having pushed into the canal, a species of blunt *tampon* of the neighboring integuments, a part of the apparatus alluded to, acts as a finger, in maintaining the tampon in place, and pressing it strongly against the anterior wall of the canal—its point of support being another piece, which to a certain extent represents the thumb of the operator. This second piece is metalic and moves at pleasure to the middle of the first, which is composed of wood. This is hollowed out with a curved canal, in which plays, like a trocar in its sheath, an armed needle, which when pushed out, opposes itself very easily to the retrograde action of the tampon in the canal. The metalic piece, instead of being smooth, is grooved in the centre, so as to permit the application of caustic to produce a slough. This cauterization, performed by means of the chloride of zinc, is applied sufficiently extensively to include the thickness of the anterior wall of the inguinal canal, and that portion of the plug which is in contact with it.

When the slough was healed, strong adhesions will have been performed between those portions of the plng, and of the canal subjected to cauterization. The apparatus is removed, when the slough falls off (from the seventh to the tenth day) the sore heals rapidly, and the nodular tissue adds force to the adhesions sought for.

"I have performed this operation," continues M. Varette, "on five patients in my wards, and I may affirm it so easily done, and so free from danger as to astonish the many students who have observed my operations. In fine, its efficacy appears to me to be certain. I hope to be able to publish a sufficient number of cases, to convince the minds of those who may read them. By that time, my method will probably have excited some prejudice against it; but this will fall before the force of the facts. What I have seen authorizes me to pronounce without hesitation, that the time is not far distant when the surgeon will radically cure a disease, which is at present treated only with palliatives."

We think that the operation of M. Varette merits careful notice on the part of the profession, though the principle upon which it is based is not new, and it is somewhat similar to the radical cure of varicocele by amputation of a portion of the scrotum.—*Philadelphia Lancet.*

